# Pattern of Recurrence and Patient Survival after Perioperative Chemotherapy with 5-FU, Leucovorin, Oxaliplatin and Docetaxel (FLOT) for Locally Advanced Esophagogastric Adenocarcinoma in Patients Treated Outside Clinical Trials

**DOI:** 10.3390/jcm9082654

**Published:** 2020-08-16

**Authors:** Torben Glatz, Rasmus Verst, Jasmina Kuvendjiska, Peter Bronsert, Heiko Becker, Jens Hoeppner, Birte Kulemann

**Affiliations:** 1 Department of Surgery, Marien Hospital Herne, Ruhr-University Bochum, Hölkeskampiring 40, 44625 Herne, Germany; Torben.Glatz@elisabethgruppe.de; 2 Center for Surgery, Department of General and Visceral Surgery, Medical Center-University of Freiburg, Hugstetter Str. 55, 79106 Freiburg, Germany; Rasmusverst@gmail.com (R.V.); jasmina.kuvendjiska@uniklinik-freiburg.de (J.K.); birte.kulemann@uniklinik-freiburg.de (B.K.); 3 Institute for SurgicalPathology, Medical Center-University of Freiburg, Breisacher Str 115A, 79106 Freiburg, Germany; Peter.Bronsert@uniklinik-freiburg.de; 4 Tumorbank Comprehensive Cancer Center Freiburg, Medical Center-University of Freiburg, 79106 Freiburg, Germany; 5 Department of Medical Oncology, Medical Center and Faculty of Medicine—University of Freiburg, Hugstetter Straße 55, 79098 Freiburg, Germany; Heiko.Becker@uniklinik-freiburg.de

**Keywords:** esophageal cancer, gastric cancer, adenocarcinoma, perioperative chemotherapy, adjuvant chemotherapy, histopathological regression

## Abstract

Background: The 5-FU, Leucovorin, Oxaliplatin and Docetaxel (FLOT) protocol provides superior oncologic results compared to other perioperative chemotherapeutic protocols for the treatment of non-metastatic esophagogastric cancer (EGAC). Survival and the pattern of recurrence of EGAC after FLOT and curative tumor resection are analyzed in a collective of patients treated outside clinical trials. Methods: Two-hundred-seventy-seven patients with EGAC (cT3-4 and/or cN+) were treated with perioperative FLOT-chemotherapy plus curative surgery between 2009 and 2018. Data were analyzed retrospectively from a prospective database. Results: Two-hundred-twenty-eight patients were included in the analysis. Postoperative in-hospital mortality was 2%. The median survival was 61–months, and median recurrence-free survival was 42 months. Multivariate analysis identified postoperative nodal status and T-stage as independent predictors of improved overall and recurrence-free survival. Administration of adjuvant chemotherapy failed to be significant for overall survival but was an independent predictor of recurrence-free survival. Recurrence occurred after a median of 9 months (range 1–46 months). Eighty-nine percent of recurrence occurred during the first 24 months. The rate of local recurrence was low. After surgery for gastric cancer, the major recurrence site was peritoneal carcinomatosis (56%), while esophageal cancer recurred mostly as metastasis to distant organs (78%). The specific site of recurrence had no impact on overall survival time. Conclusion: Real-life application of FLOT shows oncologic results comparable to clinical trials. Recurrence after FLOT and surgery for EGAC occurs predominantly early within the first two years after surgery and in the form of distant organ metastasis for esophageal tumors or peritoneal carcinomatosis for gastric tumors.

## 1. Introduction

The application of multimodal treatment protocols has a large stake in the improved prognosis of patients with locally advanced stages of esophagogastric adenocarcinoma (EGAC) by increasing surgical resectability as well as local and systemic tumor control and elimination [1,2,3,4,5].

The concept of platin-based perioperative chemotherapy has been proven to increase survival in several multicenter, randomized controlled trials and has become an increasingly popular treatment for locally advanced EGAC in specialized European cancer centers [6]. While different chemotherapy regimens were proposed at the beginning of the millennium [7,8,9], the 5-FU, Leucovorin, Oxaliplatin and Docetaxel (FLOT) protocol has recently been proven to be superior to others concerning histological tumor response and patient survival and has become the protocol of choice in numerous institutions [10,11]. We conducted this study to analyze the long-term results of FLOT obtained in applied medical care and to gain further understanding of the local and systemic effect of the FLOT regime on non-metastatic EGAC and to provide detailed information on the timing and pattern of recurrence of the tumors after resection.

## 2. Experimental Section

This study evaluates the outcome of 277 consecutive patients with histologically proven, locally advanced EGAC who were treated with FLOT and subsequent esophagectomy or gastrectomy between June 2009 and April 2018 at Medical Center University of Freiburg. Nine patients were lost to follow up and excluded from the analysis. Another 40 patients were excluded due to preoperatively detected metastatic disease. Eventually, 228 patients were included in the analysis. Informed consent was obtained from all patients before their inclusion in the cancer registry. The study was approved by the Medical Ethics Committee of the University of Freiburg (EK 253/19).

### 2.1. Pretherapeutic Work-Up

Pretherapeutic diagnostics included endoscopy with biopsies and thoraco-abdominal computerized tomography (CT) in all patients. Endoscopic ultrasound (EUS) was used routinely for staging of EGAC if technically possible. In general, lymph nodes were preoperatively classified as malignant if they were determined to be >1 cm by CT or EUS. Diagnostic laparoscopy was routinely applied in gastric cancer but not in esophageal tumors. Positron emission tomography (PET)-Scans were restricted to special cases. Indication for perioperative chemotherapy was given if therapeutic staging showed a T3 or T4 stage and/or local lymph node involvement (N+ stage).

### 2.2. Perioperative Chemotherapy (FLOT)

Starting in 2009, FLOT chemotherapy was the perioperative chemotherapy protocol of choice. Other protocols (e.g., receiving perioperative epirubicin, cisplatin and fluorouracil (ECF) [7]) were only applied if a contraindication against FLOT was present or if the patient was referred from outside after completion of neoadjuvant chemotherapy. Those patients were excluded from the analysis. FLOT protocol was carried out according to the protocol utilized in the FLOT-4 trial [11] with four 2-week neoadjuvant cycles of intravenous doxatel (50 mg/m^2^), folinic acid (200 mg/m^2^), fluorouracil (2600 mg/m^2^) (24h infusion) and oxaliplatin (85 mg/m^2^) on day 1 pre- and postoperatively. Postoperative continuance was scheduled starting 4-8 weeks after the operation with the same drug composition and dosage as preoperatively.

### 2.3. Surgery

Surgery was regularly performed 4 to 6 weeks after the end of the preoperative cycles of FLOT. The operative procedure was chosen according to tumor location. Patients with esophageal or junctional adenocarcinoma (AEG I+II) underwent esophagectomy plus proximal gastrectomy with two-field lymphadenectomy or transhiatal extended gastrectomy with lower mediastinal and modified D2-lymphadenectomy (AEG II + III). Tumors of the stomach were treated with total or subtotal (distal tumors) gastrectomy plus modified D2-lymphadenectomy.

### 2.4. Assessment of Histopathological Response to FLOT

After surgery, standardized histological workup of the specimen, including determination of the ypT- and ypN-stages, was carried out. The number of tumor-positive lymph nodes and the total number of lymph nodes removed were recorded. Histopathological regression (HPR) was identified by a pathologist according to the method described by Becker et al. [12].

### 2.5. Follow-Up

Perioperative complications were noted and graded according to Clavien-Dindo [13]. After discharge from hospital, patients were followed up at the surgical outpatient department and referred back either to the department of medical oncology or to a resident oncologist for adjuvant therapy. Follow-up was carried out at the surgical and oncological outpatient department at the multidisciplinary cancer center of Medical Center, University of Freiburg. The survival data were systematically obtained from the cancer registry of the cancer center. Data regarding postoperative chemotherapy and details of recurrence were directly obtained from the patient files.

### 2.6. Statistical Analysis

The results of our study were gained by retrospective analysis of our prospective esophagogastric database. IBM SPSS statistics, Version 23.0 was used for statistical analysis. Categorical variables were put in absolute and relative frequencies, and differences were evaluated by Chi-Square or Fisher’s exact test as appropriate. Quantitative values were expressed as medians with range, and differences were measured using the Mann–Whitney U-test. Survival was univariately analyzed by the Kaplan–Meier method with a log-rank test for the comparison of subgroups. Multivariate survival analysis was performed by the Cox proportional hazard model (forward selection strategy using a likelihood ratio statistic) including the report of relative risks and their 95% confidence intervals. A *p*-value < 0.05 was considered statistically significant.

## 3. Results

### 3.1. Patients and Tumor Characteristics

The study included 228 patients with non-metastatic EGAC treated with perioperative FLOT chemotherapy and surgery between 06/2009 and 04/2018 at the Medical Center, University of Freiburg. Seventy-eight percent of patients were male with a median age of 64 years. Ninety-seven tumors were located in the stomach (including AEG III) and 131 at the esophagus (including AEG I/II). Pretherapeutic staging classified 31 tumors as T1/2, 172 as T3 and 9 as T4 and indicated nodal disease in 153 patients. Median follow-up was 2.1 years (3.3 for censored patients and 1.2 for deceased patients). Demographic, treatment and tumor characteristics are displayed in Table 1.

### 3.2. Treatment Characteristics

Eighty-four percent of patients completed at least four preoperative cycles of FLOT, while neoadjuvant chemotherapy was incomplete in 16 percent. Patients with gastric tumors were significantly less likely to complete the neoadjuvant cycles than patients with an esophageal tumor (77% vs. 89%; *p* = 0.019). While 70% of the patients started adjuvant treatment (65% FLOT, 5% other treatment), only 38% completed the designated four cycles. Completion rate was comparable between gastric and esophageal tumors. In 13 patients, preoperative chemotherapy was extended to six and up to nine cycles of FLOT. In two patients, adjuvant chemotherapy was extended to five cycles of FLOT.

For esophageal tumors, surgery was carried out by en-bloc esophagectomy including two-field lymphadenectomy in 92% (*n* = 121) and by extended gastrectomy with modified D2-lymphadenectomy in 8% (*n* = 10) of the patients; gastric tumors were routinely resected by (transhiatal extended/subtotal) gastrectomy. Eight patients were diagnosed with distant metastases at the time of surgery, three patients received a palliative resection of the primary, and complete resection of primary and metastases was achieved in five patients.

Postoperatively, 53% of the patients developed a complication. Overall complication rate was higher in the esophageal cancer group than in the gastric cancer group (60% vs. 42%; *p* = 0.005). Serious complications requiring a surgical revision or leading to organ failure (3b or higher) were comparable in both groups (16% vs. 11%; *p* = 0.181). Postoperative in-hospital mortality was 2% (Table 1).

### 3.3. Histopathological Analysis

Pathological analysis showed a histological intestinal type in 75% of tumors and a diffuse or mixed type in 25%. The rate of diffuse and mixed types was significantly higher among gastric tumors compared to esophageal tumors (49% vs. 7%; *p* < 0.001). On final pathology, 42% (*n* = 95) were classified as nodal positive (ypN+). Histopathological complete regression was present in 14% of tumors. Thirty-two percent of tumors showed limited regression with >50% residual vital tumor cells found in the primary tumor bed in postoperative histopathological examination. Tumor regression was comparable among gastric and esophageal tumors (Table 1).

### 3.4. Pattern of Recurrence

During the follow-up period, 36% of the cohort had recurrence of the disease. Recurrence occurred after a median of 9 months (range 1–46 months). Thirty-seven percent of recurrence occurred during the first 6 months, 68% during the first year, 89% during the first 2 years and 96% during the first 3 years after surgery. While there was no difference in rate or timing of recurrence between gastric and esophageal tumors, the pattern of recurrence location was distinctively different. The rate of local recurrence was low in both groups (7% isolated local recurrence and 7% combined local recurrence and distant metastasis). After surgery for gastric cancer, the major recurrence site was peritoneal carcinomatosis (PC) (56%), while esophageal cancer recurred mostly as distant metastasis (78% of all recurrence) to liver (*n* = 11), lung (*n* = 7) or other organs (four lymphatic recurrence, three cerebral metastases, three adrenal metastases and one pleural carcinomatosis). Treatment of tumor recurrence was palliative chemotherapy in 51% or best supportive care in 24%. Surgery with curative intent was only attempted in 7% of cases (Table 2).

The subgroups of patients with gastric cancer who did not have signs of distant metastases at time of surgery (*n* = 94) had a general risk of 26% to develop a metachronous PC. Table 3 shows the risk factors for development of PC. Patients with R+ resection, diffuse tumor histology, histologically proven nodal involvement and higher pathological T-stages were at risk of developing PC. Administration of adjuvant chemotherapy had no impact on development of PC.

### 3.5. Overall and Recurrence-Free Survival

**Figure 1:**
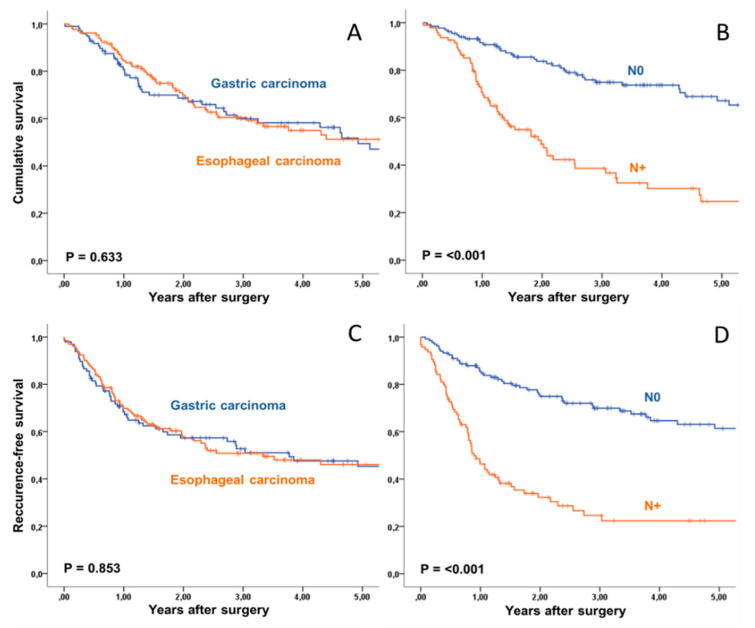
Survival in patients with esophagogastric adenocarcinoma after therapy with 5-FU, Leucovorin, Oxaliplatin and Docetaxel (FLOT) and surgical resection. Overall survival (**A**) and recurrence-free survival (**B**) of 131 patients with esophageal carcinoma and 97 patients with gastric carcinoma were similar. Nodal involvement (N+, *n* = 153) was the major predictor of overall survival (**C**) and recurrence-free survival (**D**).

The median survival for the whole collective was 61 months. The 5-year survival was 51%. Median recurrence-free survival was 42 months, and 5-year recurrence-free survival was 46%. Gender and age, type of carcinoma and tumor location as well as surgical approach and preoperative staging had no impact on survival, while American Society of Anesthesiologists (ASA) classification was prognostic for overall survival, but not recurrence-free survival. Postoperative tumor stage, postoperative nodal status, presence of distant metastasis at time of surgery, resection margin, histopathological regression grading and administration of adjuvant chemotherapy were prognostic factors for both overall and recurrence-free survival in univariate analysis (Figure 1, Table 4).

**Figure 2:**
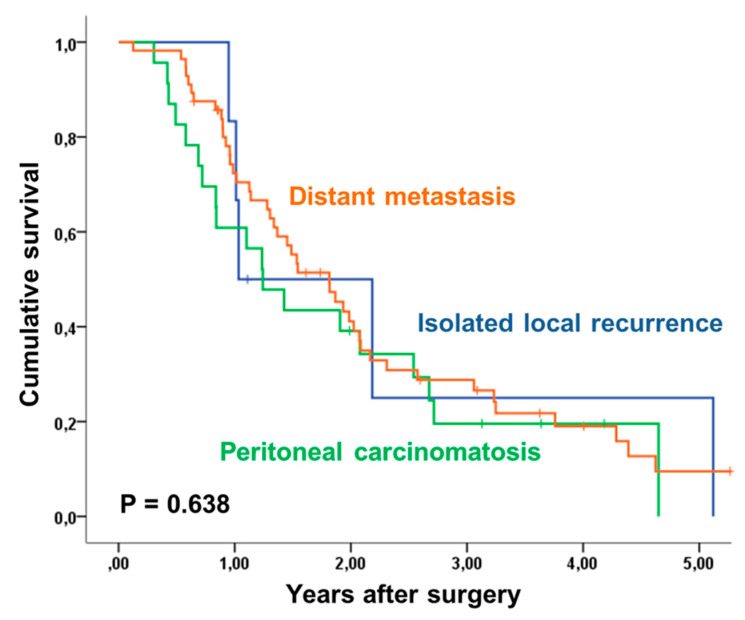
Overall Survival in 82 patients with recurrence after FLOT and surgical resection of esophagogastric adenocarcinoma: influence of recurrence site. Patients with isolated local recurrence (*n* = 6) versus distant metastasis (*n*= 53) versus peritoneal carcinomatosis (*n* = 23) expect a similar survival.

Multivariate analysis with inclusion of postoperative nodal status, tumor stage (ypT0-2 versus ypT3-4), administration of adjuvant treatment, histopathological regression, postoperative M-status, ASA-score and resection margin identified postoperative nodal status and T-stage as independent predictors of improved overall and recurrence-free survival Table 4. Administration of adjuvant chemotherapy was not significant for overall survival (*p* = 0.057) and recurrence-free survival and met the defined significance level only for the latter (RR: 1.52 (1.00–2.32) *p* = 0.050). The site of recurrence had no impact on overall survival time (*p* = 0.638; Figure 2).

## 4. Discussion

The concept of perioperative chemotherapy combined with surgery for locally advanced EGAC was proven to be effective in two randomized controlled trials, MAGIC and FFCD 9307, at the beginning of the millennium [7,8], introducing platin-based chemotherapy protocols broadly to the curative therapeutic armamentarium. The results of the most recent study, the FLOT-4 trial, show a significant survival benefit for the FLOT protocol (50 month vs. 35 month in the ECF group), introducing FLOT internationally as a standard for perioperative chemotherapy protocols for EGAC [11].

The data of this analysis show a median overall survival rate of 61 months and a recurrence-free survival rate of 42 months with FLOT and surgery, compared to a median overall survival rate of 50 months and a disease-free survival rate of 30 months in the prospective randomized FLOT-4 trial. This fact is most likely explained by the fact that the FLOT-4 trial was analyzed on an intent-to-treat basis, and the median survival estimates in our series excluded patients who were identified as having metastatic disease or local irresectable disease at the time of preoperative restaging or medical complications during chemotherapy and never made it to surgery.

Our data add a detailed insight into the pattern of recurrence after curative surgery and FLOT chemotherapy for EGAC to the results from the FLOT-4 trial. Local tumor control after surgery plus FLOT was excellent for esophageal and gastric tumors alike, with a rate of isolated local recurrence of only 3% in the overall collective. Our findings show that irrespective of excellent local tumor control, EGAC are associated with a high rate of distant tumor recurrence, necessitating a focus on effective systemic tumor elimination. Our findings are comparable to the results archived in a single high-volume referral center series utilizing the platin and taxane-based chemotherapy protocol Docetaxel, Cisplatin and Fluorouracil (DCF) [14]. In our series, patients with gastric cancer had an overall risk to develop a PC of 26% in the course of follow-up, while distant metastasis to solid organs occurred in 31% after resection for esophageal cancer.

Therefore, improving systemic tumor control has to be spotlighted in multimodal protocols of EGAC. An ongoing debate in the implementation of perioperative chemotherapy protocols concerns the impact of the adjuvant completion, considering that in our study only 40% of the patients received the full number of designated postoperative cycles. Our data show a positive effect of the additional postoperative cycles as has been shown before [15], while other studies were not able to replicate this effect [16]. Whether postoperative completion of the FLOT protocol can actually prevent any recurrence or whether it merely prolongs its occurrence remains unclear.

As an alternative to perioperative chemotherapy according to the FLOT protocol in the treatment of esophageal cancer, neoadjuvant radiochemotherapy has been proven to be superior to surgery alone in the multicenter randomized CROSS trial [17]. Adding radiation to the mix provides an excellent component of local tumor control, potentially downsizing the primary and eradicating local nodal disease. The addition of radiation to the FLOT protocol is currently investigated in the RACE trial (NCT04375605).

However, our data suggest that local tumor control provided by the FLOT protocol alone is excellent. Whether a radiation component can provide further improvement seems questionable. The results of the ESOPEC trial (NCT02509286), which is still ongoing, will provide further details on the direct comparison between the FLOT and CROSS protocols [18,19].

In the treatment of gastric cancer, studies have shown an advantage of the perioperative chemotherapy protocols to adjuvant radiochemotherapy [20], leaving these protocols as a reserve treatment. However, our data demonstrate that after surgery in curative intent for gastric cancer, the rate of peritoneal recurrence remains high despite administration of perioperative FLOT. Indeed, the administration of the adjuvant cycles did not affect the occurrence of PC. Additional adjuvant or prophylactic intraperitoneal administration of cytotoxic agents via hyperthermic intraperitoneal chemotherapy (HIPEC) has been introduced to potentially lower the rate of peritoneal recurrence [21]. A recent meta-analysis was able to establish a potential effect of prophylactic HIPEC on development of PC [22]. In clinical practice, it is not routinely used due to availability, prolonged operation time, potential additional morbidity and lack of evidence.

Furthermore, it remains unclear which patients will potentially profit from the HIPEC procedure. Our study identifies risk factors for the development of PC, which could offer a guideline to choose patients at high risk for peritoneal recurrence, who might profit from additional intraperitoneal chemotherapy. Our data also support the routine use of preoperative staging laparoscopy before neoadjuvant treatment to enable early diagnosis and tailored treatment of peritoneal metastasis [23].

The present analysis is certainly limited by its non-randomized character. Patients were not randomized to receive perioperative chemotherapy versus straight-up surgery versus different multimodal protocols (CROSS). The rate of recurrence is possibly underestimated due to incomplete follow-up data in some cases.

Taking the assets and drawbacks of our analysis into consideration, it provides a more detailed insight into the pattern of recurrence of EGAC after FLOT chemotherapy and surgery and provides a solid basis to further improve treatment and follow-up of the different patient subgroups.

## 5. Conclusions

Real-life application of FLOT shows oncologic results comparable to clinical trials. Recurrence of EGAC after FLOT and surgery predominantly occurs in the distant compartment. Recurrence generally occurs early within the first two years after surgery and in the form of distant organ metastasis for esophageal tumors or peritoneal carcinomatosis for gastric tumors.

## Figures and Tables

**Table 1:** Demographic, tumor and treatment characteristics.

	Gastric Carcinoma (N = 97)	Esophageal Carcinoma (N = 131)	Total (N = 228)
Sex			
Female	34 (35.1%)	17 (13.0%)	51 (22.4%)
Male	63 (64.9%)	114 (87.0%)	177 (77.6%)
Age in years *	64 (28-86)	63.4 (30–84)	64.0 (28–86)
ASA classification			
ASA 1–2	53 (54.6%)	68 (51.9%)	121 (53.1%)
ASA 3–4	44 (45.4%)	63 (48.1%)	107 (46.9%)
BMI in kg/m^2^ *	25.1 (16.7–37.7)	26.9 (19.0–48.7)	26.0 (16.7–48.7)
Localization			
AEG I	0 (0.0%)	69 (52.7%)	69 (30.3%)
AEG II	0 (0.0%)	62 (47.3%)	62 (27.2%)
AEG III	28 (28.9%)	0 (0.0%)	28 (12.3%)
Corpus	43 (44.3%)	0 (0.0%)	43 (18.9%)
Antrum	26 (26.8%)	0 (0.0%)	26 (11.4%)
Preoperative T stage **			
T1	1 (1.1%)	0 (0.0%)	1 (0.5%)
T2	11 (12.5%)	19 (15.3%)	30 (14.2%)
T3	72 (81.8%)	100 (80.6%)	172 (81.1%)
T4	4 (4.5%)	5 (4.0%)	9 (4.2%)
Preoperative n stage			
N0	26 (31.0%)	28 (22.8%)	54 (26.1%)
N+	58 (69.0%)	95 (77.2%)	153 (73.9%)
Lauren classification			
Intestinal	49 (50.5%)	122 (93.1%)	171 (75.0%)
Diffuse	39 (40.2%)	6 (4.6%)	45 (19.7%)
Mixed	9 (9.3%)	3 (2.3%)	12 (5.3%)
Histology			
No tumor detectable	11 (11.3%)	20 (15.3%)	31 (13.6%)
Adenocarcinoma	67 (69.1%)	107 (81.7%)	174 (76.3%)
Signet ring cell carcinoma	19 (19.6%)	2 (1.5%)	21 (9.2%)
Undifferentiated	0 (0.0%)	2 (1.5%)	2 (0.9%)
Resection margin			
R0	89 (91.8%)	126 (96.2%)	215 (94.3%)
R+	8 (8.2%)	5 (3.8%)	13 (5.7%)
Tumor regression grading			
1a	11 (11.3%)	21 (16.0%)	32 (14.0%)
1b	27 (27.8%)	26 (19.8%)	53 (23.2%)
2	30 (30.9%)	40 (30.5%)	70 (30.7%)
3	25 (25.8%)	37 (28.2%)	62 (27.2%)
4	4 (4.1%)	7 (5.3%)	11 (4.8%)
Postop. pathologic T stage			
T0	11 (11.3%)	21 (16.0%)	32 (14.0%)
T1	18 (18.6%)	19 (14.5%)	37 (16.2%)
T2	19 (19.6%)	29 (22.1%)	48 (21.1%)
T3	42 (43.3%)	59 (45.0%)	101 (44.3%)
T4	7 (7.2%)	3 (2.3%)	10 (4.4%)
Postop. pathologic n stage			
N0	60 (61.9%)	73 (55.7%)	133 (58.3%)
N+	37 (38.1%)	58 (44.3%)	95 (41.7%)
Number of removed lymph nodes *	23 (3–64)	26 (8–58)	25 (3–64)
Number of positive lymph nodes *	0 (0–34)	0 (0–29)	0 (0–34)
Postop. pathologic M stage			
M0	94 (96.9%)	126 (96.2%)	220 (96.5%)
M1	3 (3.1%)	5 (3.8%)	8 (3.5%)
Type of surgery			
Esophagectomy	0 (0.0%)	121 (92.4%)	121 (53.1%)
Gastrectomy	97 (100.0%)	10 (7.6%)	107 (46.9%)
Laparoscopic surgery	22 (22.7%)	66 (50.4%)	88 (38.6%)
Conversion rate	9 (40.9%)	3 (4.5%)	12 (13.6%)
Hospital stay in days *	11 (6–98)	15 (7–96)	13.5 (6–98)
Perioperative complications **	41 (42.3%)	79 (60.3%)	120 (52.6%)
I	7 (7.2%)	11 (8.4%)	18 (7.9%)
II	13 (13.4%)	21 (16.0%)	34 (14.9%)
IIIa	8 (8.2%)	28 (21.4%)	36 (15.8%)
IIIb	7 (7.2%)	10 (7.6%)	17 (7.5%)
IVa	2 (2.1%)	5 (3.8%)	7 (3.1%)
IVb	2 (2.1%)	1 (0.8%)	3 (1.3%)
V	2 (2.1%)	3 (2.3%)	5 (2.2%)
Neoadjuvant chemotherapy			
Complete (≥4 cycles)	75 (77.3%)	116 (88.5%)	191 (83.8%)
Incomplete (≥1 cycle–<4 cycles)	22 (22.7%)	15 (11.5%)	37 (16.2%)
Cycles of neoadjuvant chemotherapy *	4 (1–8)	4 (2–9)	4 (1–9)
Type of adjuvant chemotherapy			
None	23 (23.7%)	35 (26.7%)	58 (25.4%)
FLOT	65 (67.0%)	84 (64.1%)	149 (65.4%)
Other	6 (6.2%)	5 (3.8%)	11 (4.8%)
Not specified	3 (3.1%)	7 (5.3%)	10 (4.4%)
Adjuvant chemotherapy			
Complete (≥4 cycles)	36 (37.1%)	50 (38.2%)	86 (37.7%)
Incomplete (≥1 cycle–<4 cycles)	53 (54.6%)	69 (52.7%)	122 (53.5%)
Not specified	8 (8.2%)	12 (9.2%)	20 (8.8%)
Cycles of adjuvant chemotherapy *	4 (1–4)	4 (1–5)	4 (1–5)

* median (range); ** Data was not available for all Patients.

**Table 2:** Pattern of recurrence.

	Gastric Carcinoma (N = 97)	Esophageal Carcinoma (N = 131)	Total (N = 228)
Recurrence	36 (37%)	46 (35%)	82 (36%)
Time of recurrence after surgery (m)	9 (2–46)	9.5 (1–42)	9 (1–46)
Type of recurrence			
Local	3 (8%)	3 (7%)	6 (7%)
Local and distant metastasis	2 (6%)	4 (9%)	6 (7%)
Peritoneal carcinomatosis	20 (56%)	3 (7%)	23 (28%)
Hepatic metastasis	1 (3%)	11 (24%)	12 (14%)
Pulmonary metastasis	3 (8%)	7 (15%)	10 (12%)
Other location of metastasis	4 (11%)	11 (24%)	15 (18%)
Multiple distant metastasis	3 (8%)	7 (15%)	10 (12%)
Therapy of recurrence			
None	13 (36%)	7 (15%)	20 (24%)
Curative surgery	2 (6%)	4 (9%)	6 (7%)
Radiotherapy	0 (0%)	4 (9%)	4 (5%)
Chemotherapy	19 (53%)	23 (50%)	42 (51%)
Chemo- and radiotherapy	2 (6%)	8 (17%)	10 (12%)

**Table 3:** Risk factors for development of peritoneal carcinomatosis after FLOT and chemotherapy for gastric cancer (patients with intraoperative metastasis (*n* = 3) were excluded).

Parameter	N	Peritoneal Carcinomatosis	*P*
Total *	94	26% (*n* = 24)	
Sex			0.294
Female	33	30%	
Male	61	23%	
Age			0.320
<65	49	29%	
≥65	45	22%	
Localization			0.680
AEG III	27	22%	
Corpus	42	24%	
Antrum	25	32%	
Preoperative T Stage **			0.128
T1–T2	12	8%	
T3–T4	74	28%	
Preoperative N Stage **			0.549
N0	26	27%	
N+	56	29%	
Resection Margin			<0.001
R0	88	21%	
R+	6	100%	
Lauren Classification			<0.001
Intestinal	57	12%	
Diffuse	37	46%	
Postop. Pathologic T Stage			0.002
yT0	11	0%	
yT1	18	0%	
yT2	18	22%	
yT3	41	42%	
yT4	6	50%	
Postop. Pathologic N Stage			0.031
yN0	60	18%	
yN+	34	38%	
Histopathological Regression			0.221
1a	11	0%	
1b	26	23%	
2	30	30%	
3	23	30%	
4	4	50%	
Adjuvant Chemotherapy **			0.530
Yes	62	24%	
No	23	22%	

* Patients with esophageal tumors or presence of metastasis at time of surgery were excluded. ** Data were not available for some patients.

**Table 4:** Analysis of survival.

Univariate Analysis							
Parameter	n	Overall Survival		*p*	Recurrence-Free Survival		*p*
Total	228	51%			46%		
Sex				0.965			0.778
Female	51	49%			45%		
Male	177	51%			46%		
AGE				0.423			0.761
<65	124	54%			47%		
≥65	104	47%			45%		
ASA Classification				0.043			0.067
ASA 1–2	121	55%			53%		
ASA 3–4	107	46%			37%		
Type of Carcinoma				0.633			0.853
Esophageal Carcinoma	131	51%			46%		
Gastric Carcinoma	97	49%			45%		
Localization				0.988			0.727
AEG I	69	53%			44%		
AEG II	62	49%			49%		
AEG III	28	48%			40%		
Corpus	43	49%			47%		
Antrum	26	55%			49%		
Preoperative T Stage *				0.069			0.069
T1–T2	31	61%			60%		
T3–T4	181	49%			44%		
Preoperative N Stage *				0.742			0.972
N0	54	46%			45%		
N+	153	53%			47%		
Type of Surgery				0.705			0.928
Esophagectomy	121	51%			45%		
Gastrectomy	107	50%			46%		
Resection Margin				<0.001			<0.001
R0	214	53%			48%		
R+	14	16%			13%		
Histopathological Regression				<0.001			<0.001
1a	32	68%			69%		
1b	53	68%			62%		
2	70	51%			44%		
3	62	36%			30%		
4	11	9%			9%		
Postop. Pathologic T Stage				<0.001			<0.001
yT0	32	68%			69%		
yT1	37	74%			72%		
yT2	48	62%			48%		
yT3	101	36%			33%		
yT4	10	0%			0%		
Postop. Pathologic N Stage				<0.001			<0.001
yN0	133	67%			61%		
yN+	95	25%			22%		
Postop. Pathologic M Stage				0.023			0.002
M0	220	51%			47%		
M1	8	22%			16%		
Adjuvant Chemotherapy *				0.024			0.025
Yes	149	54%			49%		
No	58	42%			38%		
Recurrence				0.638			
Isolated Local Recurrence	6	25%					
Peritoneal Carcinomatosis	23	0%					
Distant Metastasis	53	9%					
MULTIVARIATE ANALYSIS							
	Overall Survival				Recurrence-Free Survival		
Parameter	RR	95%-CI	*p*		RR	95%-CI	*p*
Postop. N Stage (YPN0/YPN+)	2.53	1.54–4.17	<0.001		2.87	1.80–4.57	<0.001
Postop. T Stage (YPT0-2/YPT3–4)	2.06	1.22–3.46	0.006		1.77	1.10–2.84	0.018
Postop. Chemotherapy (Y/N)	---	---	0.057		1.52	1.00–2.32	0.05
Histopath. Regr. (<10/10–50 > 50%)	---	---	0.110		---	---	0.106
Postop. M Stage (YM0/YM+)	---	---	0.204		---	---	0.231
ASA (1–2/3–4)	---	---	0.126		---	---	0.273
Resection Margin (R0/R+)	---	---	0.400		---	---	0.685

Univariate analysis by Kaplan–Meier method with log-rank test for the comparison of subgroups/, Multivariate survival analysis by the Cox proportional hazard model (forward selection strategy using a likelihood ratio statistic) including the report of relative risks and their 95% confidence intervals. * Data not available for some patients.
